# Assessment of Sleep Duration Based on Ankle and Wrist Actigraphy in Hospitalized Older Patients.

**DOI:** 10.1093/geroni/igab046.2396

**Published:** 2021-12-17

**Authors:** Juliana Smichenko, Tamar Shochat, Nurit Gur-Yaish, Anna Zisberg

**Affiliations:** 1 University of Haifa, Haifa, Hefa, Israel; 2 University of Haifa, University of Haifa, HaZafon, Israel; 3 Oranim Academic College of Education, Haifa, HaZafon, Israel; 4 University of Haifa, Haifa, HaZafon, Israel

## Abstract

Poor sleep at time of hospitalization is associated with undesirable outcomes. Most studies performed in the hospital assess sleep by self-report, while only few rely on actigraphy. Although wrist actigraphy is commonly used for sleep assessment in field studies, in-hospital assessment may be challenging and cumbersome due to other more necessary monitoring devices that are often attached to patients’ upper limbs, that may in turn affect interpretation of wrist activity-data. Placement on the ankle may be a viable solution. In this pilot study, we aimed to compare total sleep time (TST) using concomitant wrist and ankle actigraphy as well as self-report. Twenty-one older adults (65+) hospitalized in medical units wore ankle and wrist actigraphy devices and subjectively estimated their TST for an average of (2.15±1.01) nights. A total of 45 nights were available for analysis. Average TST in minutes was 332.06±81.58, 427.05±97.74 and 374.28±124.96 based on wrist, ankle, and self-report, respectively. Repeated measure mixed models analysis was performed adjusting for age, gender, and sleep medications. TST was significantly lower using wrist compared to ankle actigraphy (F(2,102)=7.63, p=0.0008), and both were not different from self-report. No significant within subjects variation and no interaction between device and repeated measures were found. Despite differences between ankle and wrist assessments, all three provide consistent TST estimation within subjects. Self-report provides a stable and accessible assessment of TST, representing a good approximation of ankle and wrist actigraphy. Findings provide preliminary support for the use of ankle actigraphy for sleep assessment in hospital settings.

